# Home Monitoring in Interstitial Lung Disease: Protocol for a Real-World Observational Study

**DOI:** 10.2196/65339

**Published:** 2025-06-12

**Authors:** Marium Naqvi, Rebecca Borton, Sarah Lines, Joanne Dallas, Jessica Mandizha, Howard Almond, Colin Edwards, Wendy Adams, Michael Gibbons, Anne-Marie Russell, Alex West

**Affiliations:** 1 Department of Respiratory Medicine Guy’s and St Thomas Hospitals NHS Trust London United Kingdom; 2 Peter Gorer Department of Immunobiology King's College London London United Kingdom; 3 patientMpower Ltd Dublin Ireland; 4 Interstitial Lung Disease Unit Respiratory Medicine Royal Devon University Healthcare NHS Foundation Trust Exeter United Kingdom; 5 Exeter Respiratory Innovations Center University of Exeter Exeter United Kingdom; 6 EPIC Group University of Exeter Exeter, Devon United Kingdom; 7 Action for Pulmonary Fibrosis Peterborough United Kingdom; 8 College of Medicine and Health School of Health Sciences, Medical School University of Birmingham Birmingham United Kingdom; 9 University of Exeter Exeter United Kingdom

**Keywords:** remote monitoring, interstitial lung disease, spirometry, real-world, multicenter, observational study, home monitoring

## Abstract

**Background:**

Interstitial lung diseases (ILDs), a group of parenchymal lung disorders, present with varying degrees of inflammation and fibrosis, which lead to symptoms such as progressive breathlessness, impaired quality of life (QoL), and reduced life expectancy. Patients with ILD can experience a sudden worsening of their condition, known as an acute exacerbation, which is associated with inappropriate hospital admissions, concomitant National Health Service (NHS) costs, impaired QoL, and high mortality. The heterogeneity of ILDs, the unpredictability of acute exacerbations, and significant variation in disease progression and response to treatment present numerous management challenges. Standard care typically involves 3-6 monthly hospital outpatient visits to monitor disease and assess response to treatment. Home monitoring with remote review of spirometry, pulse oximetry, and patient-reported measures offers an alternative approach to in-person clinic review and laboratory-based physiological measurements. Clinical trials indicate home monitoring of patients with ILD is acceptable, and results correlate with laboratory-based pulmonary function tests (PFTs). The impact of implementing home monitoring for patients with ILD in a real-world setting is not well understood.

**Objective:**

We aim to evaluate the safety, effectiveness, and acceptability of home monitoring with standard care in the management of patients with ILD.

**Methods:**

This study has been registered as a quality improvement project at Guy’s and St Thomas’ NHS Foundation Trust (reference 13660) and Royal Devon University Healthcare NHS Foundation Trust (reference 24-1378). The project has been co-designed by the steering group, including clinicians, researchers, technology partners, a patient advocacy charity, and patients diagnosed with ILD. Patients who meet the inclusion criteria will be provided a handheld spirometer, pulse oximeter, and access to patientMpower, an electronic health app, on their smart devices and followed up for 12 months. All participants will be asked to complete at least once weekly home spirometry and pulse oximetry measurements and 3 monthly patient-reported measures, including outcome, engagement, and experience measures, using the patientMpower app. Results will be available to the clinicians in real time and used to monitor disease progression, symptoms, and QoL, and to assess treatment response.

**Results:**

This study was funded by NHS Digital in September 2021. Patient recruitment and data collection started in March 2022. By January 2024, 186 patients were enrolled. All patients will have home monitoring for at least 12 months. Results are expected to be published at the end of 2025.

**Conclusions:**

We hypothesize home monitoring will be safe, effective and acceptable for patients with ILD and result in a 50% reduction in routine laboratory-based pulmonary function tests and in-person clinic consultations.

**International Registered Report Identifier (IRRID):**

DERR1-10.2196/65339

## Introduction

### Interstitial Lung Disease

Interstitial lung diseases (ILDs), a group of parenchymal lung disorders, present with varying degrees of inflammation and fibrosis [1]. Idiopathic pulmonary fibrosis (IPF) is the archetypal, invariably progressive, fibrotic lung disease characterized by a decline in lung function, worsening breathlessness, impaired quality of life (QoL), and reduced life expectancy [2,3]. Other ILD subtypes may develop into a progressive fibrotic phenotype despite conventional therapies and are described as progressive pulmonary fibrosis (PPF) [4]. Shared mechanistic and clinical features have been identified between PPF and IPF such as worsening respiratory symptoms, decline in lung function, and premature mortality [5-12]. The heterogeneity of ILDs, the unpredictability of acute exacerbations, and significant variation in disease progression and response to treatment present numerous challenges in diagnosis and management.

The gold standard for ILD diagnosis requires a multidisciplinary team, preferably an interdisciplinary team, an approach where experts, including respiratory physicians, rheumatologists, thoracic radiologists, pathologists, and ILD specialist nurses, review clinical symptoms, exposure history, serology, and radiology [4]. Frequent monitoring, including assessment of symptoms, functional capacity, and physiological measurements, is recommended to enable the identification of disease progression, assess response to treatment, and inform ongoing management [4,13]. The frequency of follow-up is determined by the severity of disease progression, deterioration in QoL, and available resources [4,14]. In-person clinic review often places a significant physical burden on patients due to exertional breathlessness, oxygen dependency, infection risk, work commitments, and the need for carer support. In the United Kingdom, many patients travel long distances with inadequate transport provision to reach ILD specialist services. Consequences include delayed follow-up and increased costs to the patient, the National Health Service (NHS), and the environment [15].

### Home Monitoring

Clinical trials have demonstrated that home spirometry is acceptable to patients with ILD [16-18]. Home spirometry correlates with clinical spirometry and is thought to be effective in detecting disease progression in patients with ILD [19,20]. The widespread increase in societal digitization, including smartphone utilization, has enabled the development of digital care pathways. The COVID-19 pandemic further accelerated the utilization of home monitoring devices, such as pulse oximeters, to support admission avoidance or early discharge from the hospital, shifting the emphasis to supporting the delivery of care closer to home [21,22]. Home monitoring of patients with ILD is not yet part of standard care, despite its perceived utility. This may be due to organizational factors, clinician workload, funding, patient factors including the ability to adopt digital technology, perceived risk to psychological well-being, and concerns regarding the correlation of home and laboratory-based pulmonary function tests (PFTs) [18,23,24].

Home monitoring, including spirometry, pulse oximetry, and patient-reported measures (PRMs), with remote review offers an alternative approach to in-person clinic review and laboratory-based PFTs for patients with ILD. It has the potential to support early identification of disease progression or acute exacerbation, assess response to treatment, enhance QoL, and alleviate health care burden [23]. Evidence of the long-term safety, effectiveness, and acceptability of home monitoring, including assessment of the impact on clinical outcomes, resource use, and patient satisfaction is needed to optimize this approach to patient care. We aim to assess if home monitoring is safe, effective, and acceptable for patients with ILD when offered in addition to standard care. We hypothesize home monitoring will lead to a 50% reduction in routine laboratory-based PFTs and in-person clinic consultations.

## Methods

### Study Design

This study has been co-designed using the ObsQual (Observational and Qualitative Study Protocol Reporting) checklist with a steering group of clinicians, researchers, technology partners, a patient advocacy charity, and patients with ILD [25]. All participants will be offered a portable handheld Spirobank Smart MIR spirometer and Nonin 3230 Bluetooth pulse oximeter. These will be linked to patientMpower, an electronic health app, downloaded to the patient’s smart device. The recruiting clinician will counsel the patient, provide written information, and gain informed patient consent. Trained clinicians will provide patient training on the use of the home spirometer, pulse oximeter, and completion of PRMs, in person or virtually.

**Figure 1:**
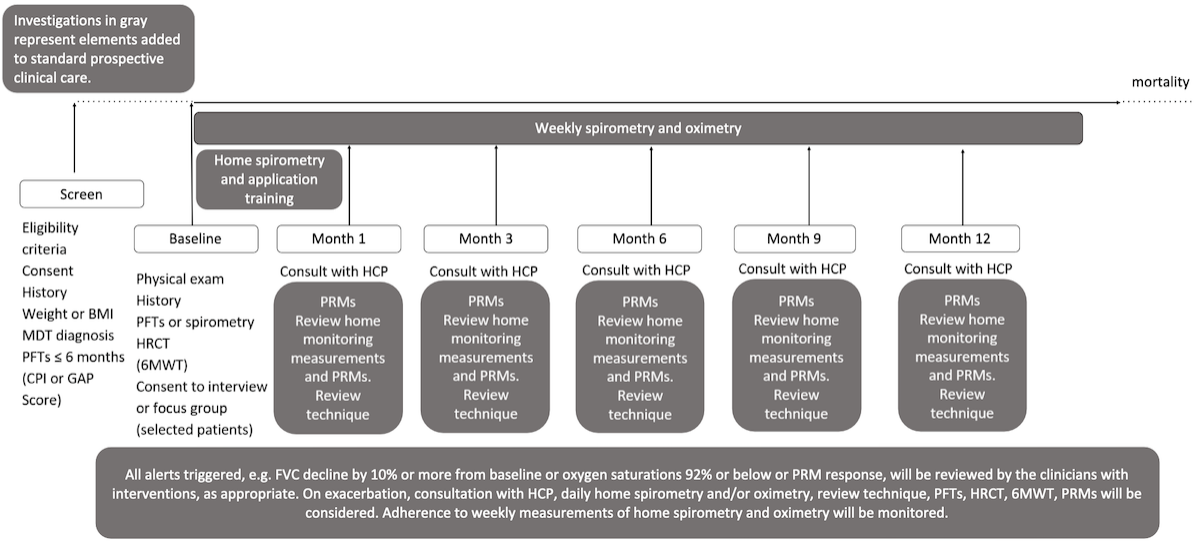
Interstitial lung disease supported self-management schematic. PFTs included FVC, forced expiratory volume, diffusing capacity of the lungs for carbon monoxide, carbon monoxide transfer coefficient, and partial pressure of oxygen in the alveoli. PRMs include idiopathic pulmonary fibrosis patient-reported outcome measure, modified Medical Research Council dyspnea score, EQ-5D-5L, cough visual analogue scale, Global Rate of Change Questionnaire, SpiroQ (home spirometry satisfaction questionnaire), 9-item Patient Health Questionnaire, 7-item General Anxiety Disorder, Porter-Novelli, and modified patient-reported experience measure for patients with rheumatoid arthritis. 6MWT: 6-minute walk test; CPI: composite physiologic index; FVC: forced vital capacity; GAP: gender-age-physiology; HCP: health care professional; HRCT: high-resolution computed tomography; MDT: multidisciplinary team; PFT: pulmonary function test; PRM: patient-reported measure.

Patients will be asked to record spirometry and pulse oximetry measurements at least once weekly, and a series of PRMs at baseline and at months 3, 6, 9, and 12. These include the idiopathic pulmonary fibrosis patient-reported outcome measure (IPF-PROM), modified Medical Research Council dyspnea scale (mMRC), EQ-5D-5L, cough severity visual analog scales (VAS),Global Rating of Change Questionnaire (GRCQ), 9-item Patient Health Questionnaire (PHQ-9), 7-item General Anxiety Disorder (GAD-7), and experience and engagement measures, including Porter-Novelli, a home spirometry satisfaction questionnaire (SpiroQ), and the interstitial lung disease patient-reported experience measure (modified from the rheumatoid arthritis patient-reported experience measure) [26-34]. These PRMs have been used and validated in IPF and PPF populations, except for SpiroQ, which was co-designed with the steering group to support us in measuring patient satisfaction with home monitoring. EQ-5D-5L is a health utility measure that will enable health economic evaluation [35]. The timeline of measurements is demonstrated in Figure 1. All data recorded via the app will be visible to the patient and the clinicians in real-time via a secure browser-based portal.

The registering clinician will be responsible for setting thresholds for a ≥10% decline in absolute forced vital capacity (FVC) and oxygen saturation <94%, according to the individual’s baseline measurement [34]. If measurements fall below the threshold, alerts will be sent to the clinical team via the browser-based portal. The clinical team will review daily alerts during weekday working hours (Monday to Friday, 9 AM-5 PM). Each review will consider measurements, quality of the measurements, trends, changes from baseline, and contributing factors. Patients may require follow-up telephone or in-person clinic assessment to guide management decisions, for example, with repeat high-resolution computed tomography (HRCT), 6-minute walk test, long-term oxygen therapy assessment, and laboratory-based PFTs. Patients will continue to receive standard care as per their local clinical protocols.

### Setting

Patients will be recruited from ILD specialist clinics at Guy’s and St Thomas’ NHS Foundation Trust (GSTT) and Royal Devon University Healthcare NHS Foundation Trust (RDU), from March 2022 to January 2023. All patients will have 12 months of home monitoring. Alerts will be managed by ILD specialist clinicians at each site. All clinical data will be collected on the browser-based portal and in an electronic case report form within secure Microsoft Office software only accessible to participating clinicians.

### Participants

A total of 200 patients will be recruited from 2 ILD specialist services in the United Kingdom to achieve robust and comprehensive insights from a diverse patient population. Adhering to the inclusion and exclusion criteria, ILD specialist clinicians will recruit adults aged 18 years and older, with a tertiary center multidisciplinary diagnosis of ILD confirmed by HRCT and expectation of variability in progression. Patients will need to be able to perform spirometry and achieve a baseline FVC >45% predicted value within 6 months of recruitment. They will need access and the ability to use a smart device with internet and email. A budget will be available to provide smart devices and internet connectivity to patients who do not have access. Patients will need to be able to communicate with the team, understand written instructions, and complete PRMs. Patients will not be able to participate if they have a life expectancy of less than 6 months determined by clinical judgment, contraindications to spirometry as per Association for Respiratory Technology and Physiology guidelines, upper or lower respiratory tract infection within 4 weeks, acute exacerbation within 4 weeks, and active cancer diagnosis or life-limiting malignancy [36].

All patients will have 3 monthly virtual reviews with an ILD specialist clinician. Home monitoring measurements will be reviewed. Patients will be asked to report any upper or lower respiratory tract infections, acute exacerbations, accident and emergency presentations, or hospital admissions in the previous 3 months. Responses will be documented on the browser-based portal and electronic case report form. Daily alerts will be reviewed and addressed by the clinical team. Alert management will be documented on the browser-based portal and electronic case report form. Patients who miss weekly measurements for 3 weeks or choose to withdraw from home monitoring will be contacted, and the reason for stopping will be documented on the browser-based portal.

### Variables

The variables to be measured and how they will be measured are outlined in Table 1.

**Table 1:** Outcome measures.

		Measurement tools
**Primary outcome measures**		
	Potential to detect disease progression, acute exacerbation, or response to treatments enabling prompt review of patients and access to appropriate therapies compared with standard care	Browser-based portal: Number of patients with ≥5%-10% decline in FVC^a^ Time to ≥5%-10% decline in FVC Number of patients diagnosed with progressive pulmonary fibrosis who become eligible for antifibrotic treatment PRM^b^ changes in score Number of patients who start or change their prescription for oxygen therapy Time to titrate or start oxygen therapy Electronic case report form: Number of acute exacerbations and how they were managed Interventions made (type and number)
	Impact on the number of in-person clinic consultations with ILD^c^ clinicians compared with standard care	Electronic case report form: (data comparison with 2019 to 2020): Number of F2F^d^ consultations Number of telephone consultations Number of video consultations
	Impact on the number of laboratory-based pulmonary function tests compared with standard care	Electronic case report form (data comparison with 2019 to 2020): Number of F2F consultations Number of telephone consultations Number of video consultations
	Real-world patient acceptability of home monitoring program, including spirometry, oximetry, and PRMs	Browser-based portal: Frequency of patient recording of key measurements (eg, spirometry, pulse oximetry, PRMs) Duration (minutes) of regular patient engagement with the application Patient engagement and experience measure ratings Electronic case report form: Patients not eligible or declined participation and reason
**Secondary outcome measures**		
	Impact on clinician time and experience compared to standard care	Browser-based portal: Clinician time spent on the browser-based portal Electronic case report form: Time taken to address each alert
	Correlation of PRMs with patient symptoms	Electronic case report form: Review symptoms and measurements every 3 months
	Number of patient requests for technical support	Browser-based portal: Number and length of unique interactions with the technical team
	Impact on the number of ILD-related hospital admissions and duration of inpatient stay	Electronic case report form: Review admissions every 3 months
	Impact on patient access to ILD specialized services	Browser-based portal: Completion of PRMs Electronic case report form (data comparison with defined standard care, eg, 3-4 per year, and 2019 to 2020): Number of interactions with the ILD team
	Correlation between home monitoring and patient satisfaction	Browser-based portal: PRMs Duration and frequency of patient app use
	Impact on travel costs to the patient, NHS^e^, and environment	Electronic case report form: Postcode and usual mode of travel at registration Cost of mode of transport (car mileage 45p per mile) Dependent on carer support

^a^FVC: forced vital capacity.

^b^PRM: patient-reported measure.

^c^ILD: interstitial lung disease.

^d^F2F: face-to-face.

^e^NHS: National Health Service.

### Bias

We will reduce bias by recruiting patients from a diverse geographical patient population across 2 ILD specialist services in the south of England. Patients will be recruited from the ILD specialist clinics using a randomized approach. In addition, we will undertake sensitivity analyses to account for potential biases and uncertainties, ensuring the robustness of the findings.

### Study Size

The sample size for this study is pragmatic, based on local populations within the clinical service and feasibility within financial constraints.

### Quantitative Variables

Statistical analysis will include patient demographic data, including age, gender, ethnic background, smoking status, occupation and geographical location, measurements and PRM responses, number and type of alerts and interventions, number and mode of clinic consultations, and investigations.

### Statistical Methods

A data management standard operating protocol will be co-developed considering the range and variation in data types. The steering group will meet fortnightly to check data for quality and consistency. Missing data will be identified, inconsistencies resolved, and data completed where possible. All end points will be summarized for descriptive statistics display. Values will be expressed as mean (SD) or median and range. Comparison of baseline home and hospital spirometry will use the Bland-Altman method. The rate of FVC change will be calculated using all available values using linear regression analysis without imputation of missing values. The rate of change in FVC will be presented as the percentage change relative to baseline values. For home-based spirometry, the baseline value will be calculated according to the mean of the values obtained at baseline and week 2. Survival analyses will be analyzed using Kaplan-Meier plots if we are defining disease progression as death or a >10% decline in home-based FVC at 12 months. A *P* value of <.05 will be considered statistically significant.

### Ethical Considerations

The study design and protocol have been registered and peer-reviewed as a quality improvement project in the 2 participating centers (GSTT reference 13660 and RDU reference 24-1378), as per local Trust policies. All patients will provide informed consent to participate in home monitoring and be included in data analysis. All patients will be able to opt out at any time and return to standard care. No remuneration will be offered as home monitoring is being offered in addition to standard care. Data will be stored for the duration of the project and for 8 years after the end. All data will be handled in accordance with the Data Protection Act 2018. A data processing agreement is in place at both Trusts to support this data being shared under the Data Protection Act. All data will be anonymized and grouped for presentation and publication.

## Results

This study was funded by NHS Digital in September 2021. Patient recruitment and data collection started in March 2022. By January 2024, we recruited 186 patients. All patients will have home monitoring for at least 12 months. Data analysis is ongoing, and results are expected to be published at the end of 2025. No significant safety concerns are anticipated with any measurements carried out as part of this study. In the case of adverse events, the established clinical safety procedures, investigation protocols, and reporting guidelines for each Trust will be adhered to, ensuring thorough and consistent management in accordance with established guidelines. The approach to care outlined in this protocol signifies a potentially pivotal moment in the transformation of health care delivery to patients with ILD.

## Discussion

### Previous Work

The incidence and prevalence of PPF and IPF are increasing [37,38]. A recent real-world study suggested that up to 20% of patients treated for PPF had advanced disease [37]. This is associated with productivity loss, increased health care costs, and reduced QoL [39]. Detecting and treating acute exacerbations early is likely to positively impact health care resource consumption, and direct or indirect costs, and enable informed choice relating to the place of death. Previous studies have demonstrated good adherence and feasibility of home monitoring in ILD, in clinical trial settings, and when observed in study populations [16,17,20,40-43]. A systematic review found that home monitoring presented an opportunity for early detection of disease progression or acute exacerbation [18]. The COVID-19 pandemic accelerated the utilization of home monitoring and supporting delivery of care closer to home [21,22].

### Principal Findings

We hypothesize that the findings of this study will demonstrate that home monitoring is safe, effective, and acceptable to patients with ILD, in a real-world setting. The results will provide the evidence required to inform the adoption of home monitoring for ILD clinical care and a digital care pathway. In addition to the primary outcomes, this study may inform a health economic evaluation and the impact of digital and health literacy on adherence. This study will also investigate differences in terms of patient characteristics and demographics in 2 diverse regions of the United Kingdom. We anticipate this research will provide valuable evidence to inform the development of guidelines relating to digital care pathways for patients with IPF and PPF, and decision-making by policy makers and integrated care boards.

### Strengths and Limitations

The strengths of this study include the implementation of home monitoring in a real-world setting, across 2 NHS Trusts with diverse geographical and socioeconomic patient representation. Home monitoring will include spirometry, oximetry, and PRMs to assess changes in patient symptoms, disease, and response to treatment. We will undertake long-term home monitoring of a chronic lung condition compared to short-term acute virtual wards. The limitations of this study include real-world practice, which may lead to inconsistencies in data collection across the 2 NHS sites.

### Future Directions

This study will inform the adoption of home monitoring in ILD, a long-term chronic condition, in the NHS. It will identify the knowledge gaps and barriers to implementing home monitoring in real-world practice. We anticipate that further research questions will arise, informing future work.

### Dissemination

Our findings will be disseminated through communications of results to participants, people living with fibrotic lung disease, and their stakeholders, including caregivers, health care professionals, and the scientific community. Specific activities include dissemination through publication, including at national and international respiratory conferences and open-access peer-reviewed publication of findings. Further, we will communicate our findings through the internet and social networks associated with our patient charity and technology partners.

### Conclusion

Despite limitations, home monitoring offers an opportunity to deliver personalized, home-centered care. Multimodal care delivery is required to meet the needs of this patient population, and digital care may lead to improved engagement with health care, adherence to treatment, and improved QoL.

## Abbreviations

FVC: forced vital capacity
GSTT: Guy’s and St Thomas’ NHS Foundation Trust
ILD: interstitial lung disease
IPF: idiopathic pulmonary fibrosis
NHS: National Health Service
ObsQual: Observational and Qualitative Study Protocol Reporting
PFT: pulmonary function test
PPF: progressive pulmonary fibrosis
PRM: patient-reported measure
QoL: quality of life
RDU: Royal Devon University

## Data Availability

All data generated or analyzed during this study will be included in the published article (and its supplementary information files), where possible.
